# Cross-cultural validity of the Pulmonary Embolism Quality of Life questionnaire in the quality of life survey after pulmonary embolism: A Persian-speaking cohort

**DOI:** 10.1016/j.rpth.2023.100145

**Published:** 2023-04-01

**Authors:** Kasra Mehdizadeh, Maryam Mohseni Salehi, Jamal Moosavi, Bahram Mohebbi, Frederikus A. Klok, Behnood Bikdeli, Omid Shafe, Hamidreza Pouraliakbar, Azin Alizadehasl, Melody Farrashi, Raheleh Kaviani, Farzaneh Mehrvarz, Farid Rashidi, Hamed Talakoob, Hooman Bakhshandeh, Parham Sadeghipour

**Affiliations:** 1 Rajaie Cardiovascular Medical and Research Center, Iran University of Medical Sciences, Tehran, Iran; 2 Cardiovascular Intervention Research Center, Rajaie Cardiovascular Medical and Research Center, Iran University of Medical Sciences, Tehran, Iran; 3 Department of Medicine-Thrombosis and Hemostasis, Leiden University Medical Center, Leiden, Netherlands; 4 Center for Thrombosis and Hemostasis, University Medical Center Mainz, Mainz, Germany; 5 Cardiovascular Medicine Division, Brigham and Women’s Hospital, Harvard Medical School, Boston, Massachusetts, USA; 6 Thrombosis Research Group, Brigham and Women’s Hospital, Harvard Medical School, Boston, Massachusetts, USA; 7 YNHH/Yale Center for Outcomes Research and Evaluation (CORE), New Haven, Connecticut, USA; 8 Cardiovascular Research Foundation (CRF), New York, USA; 9 Echocardiography Research Center, Rajaie Cardiovascular Medical and Research Center, Iran University of Medical Sciences, Tehran, Iran; 10 Tuberculosis and Lung Diseases Research Center, Tabriz University of Medical Sciences, Tabriz, Iran

**Keywords:** pulmonary embolism, quality of life, patient-reported outcome measures, surveys and questionnaires, psychometrics, reproducibility of results

## Abstract

**Background:**

The Pulmonary Embolism Quality of Life (PEmb-QoL) questionnaire is the first disease-specific scale for assessing the quality of life in patients with a history of pulmonary embolism (PE).

**Objectives:**

To assess the cross-cultural validity and reliability of the disease-specific PEmb-QoL questionnaire.

**Methods:**

The Persian version was prepared through the forward and backward translation of the English questionnaire. Six months after the diagnosis of acute PE, consecutive Persian-speaking patients were asked to complete the PEmb-QoL, the generic 36-item Short Form (SF-36) questionnaires and undertake a 6-minute walk test (6MWT). Acceptability was assessed via item missing rate, reproducibility by the test-retest method, and internal consistency reliability by Cronbach’s α and McDonald’s ω coefficients. Convergence validity was assessed using the Spearman rank correlation between scores of PEmb-QoL, SF-36, and 6MWT. The questionnaire structure was evaluated through exploratory factor analysis.

**Results:**

Ninety-six patients with a confirmed diagnosis of PE completed the questionnaires. The Persian version of PEmb-QoL had good internal consistency (α = 0.95, 3-factor ω = 0.96), inter-item correlation (0.3–0.62), item-total correlation (0.38–0.71), reproducibility (test-retest ICC with 25 participants = 0.92–0.99), and good discriminant validity. Convergence validity was confirmed by the moderate-to-high correlations between PEmb-QoL and SF-36 scores, and a good correlation between the “limitation in daily activities” dimension of the PEmb-QoL questionnaire and 6MWT results. Exploratory factor analysis suggested a 3-component structure with functional (items 1h, 4b-5d, 6, 8, 9i, and 9j), symptoms (1b-h, 7, and 8), and emotional (5a, 6, and 9a-h) components.

**Conclusion:**

The Persian version of the PEmb-QoL questionnaire is valid and reliable for measuring the disease-specific quality of life in patients with PE.

## Essentials

• Pulmonary embolism (PE) has been shown to impact different aspects of the quality of life (QoL).
• PE quality of life (PEmb-QoL) is a disease-specific survey for patients with PE.
• Cross-cultural validity and reliability of PEmb-QoL were confirmed in a Persian-speaking cohort.
• Minor adjustments in dimensions and their constituent items were required.

## Graphical abstract

**Figure:**
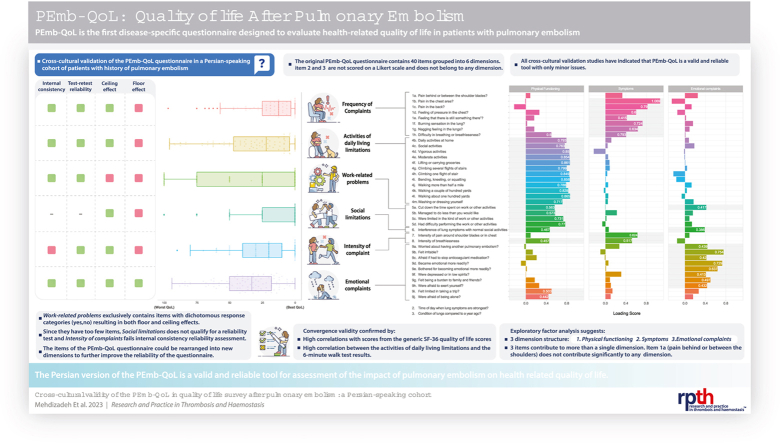

## 1 Introduction

The post-pulmonary embolism (PE) syndrome is defined as new or progressive dyspnea, exercise intolerance, and/or impaired functional or mental status after at least 3 months of adequate anticoagulation following acute PE, which cannot be explained by other (preexisting) comorbidities [[1], [2], [3]]. Although the extent of the disability varies in different populations [4], nearly half of the patients will have persistent dyspnea, with 11% suffering from moderate-to-severe functional limitations [5]. The chronic complications of acute PE are also taxing on one’s psychological and social status. The long-term risk of purchasing psychotropic drugs is substantially increased in adolescents with a history of PE, with more than a quarter of the previously employed patients not returning to work even one year after the initial PE episode [[6], [7], [8]].

Previous statements highlight that the chronic complications actually experienced following acute PE go well beyond the implications of clinically measured outcomes such as RV dysfunction and pulmonary hypertension [4,9,10]. Therefore, valuable information concerning patients’ well-being would be lost if information acquisition is channeled only through the narrow scope of clinical and paraclinical evaluations [11]. Direct formal inquiry of patients’ experiences through adequately validated patient-reported outcome (PRO) measures also confers the advantage of negating interobserver variability and is, thus, potentially more reliable than when such experiences are informally inquired, interpreted, and reported by third parties [12].

Health-related quality of life is a multidimensional construct encompassing one’s self-perception of, at a minimum, physical, emotional, and social well-being [13]. It is usually assessed using both generic and specific PRO questionnaires. Whereas generic questionnaires allow comparison between different populations irrespective of their underlying conditions, condition-specific questionnaires are more sensitive to changes in the frequency and severity of specific outcomes, making them the instrument of choice for evaluating the impacts of therapeutic and rehabilitation strategies [14].

Pulmonary embolism quality of life (PEmb-QoL) questionnaire is the first and currently the only disease-specific PRO instrument for patients with a history of PE. The questionnaire was developed in Dutch, cross-culturally validated in several other languages, and is part of the recently developed core set of outcome measures for patients with PE [11,[15], [16], [17], [18], [19], [20]]. Our study aimed to prepare the first Persian-translation PEmb-QoL, provide an ad hoc evaluation of its psychometric properties, and adjust its structure based on a Persian-speaking PE patient population in Iran.

## 2 Methods

Our study aimed to prepare the Persian version of PEmb-QoL based on the English version and then evaluate its psychometric properties (acceptability, reliability, and validity) as to whether it is an appropriate instrument for measuring PE-related quality of life.

### 2.1 Study setting and participants

All Persian-speaking surviving patients with records in the Pulmonary Emboli Registry of Rajaie Cardiovascular Medical and Research Center (RHC-PE) between September 2015 and August 2018 were invited via phone calls to participate in the present prospective study. The RHC-PE registry is an all-comers cohort of patients with a confirmed diagnosis of PE [21]. Briefly, PE was diagnosed mainly via computed tomography pulmonary angiography. The baseline demographic and clinical characteristics were retrieved from the RHC-PE registry. All the information gathered was recorded during the index hospital admission. The risk stratification of the study population was performed according to the European Society of Cardiology guidelines for the diagnosis and management of acute PE [22]. The study protocol was approved by the Ethics Committee of the Rajaie Cardiovascular Medical and Research Center, and signed informed consent was obtained from all the study participants.

### 2.2 Data collection and follow-up

Each patient was scheduled for a structural 6-month follow-up program composed of detailed transthoracic echocardiography and a 6-minute walk test (6MWT). During the program, a QOL assessment was performed using the disease-specific PEmb-QoL and the generic 36-item Short Form (SF-36) survey over an interview session assisted by an experienced nurse.

### 2.3 Instruments

#### 2.3.1 36-Item Short Form (SF-36) survey

SF-36 is a widely used, generic, health-related QOL questionnaire validated in many disease cohorts and languages, including Persian [23]. The questionnaire covers 8 dimensions grouped into 2 summary components: physical and mental [24,25]. The detailed structure of the SF-36 questionnaire is depicted in Supplementary Figure S1. In this study, SF-36 scores were calculated according to the RAND scoring instructions [26].

#### 2.3.2 PEmb-QoL questionnaire

Modeled based on the generic SF-36 and the disease-specific quality of life after acute DVT (VEINES-QOL/Sym) questionnaire, the PEmb-QoL was designed to assess disease-specific health-related QOL in patients with a history of acute PE [15]. It contains 40 items, 38 of which are on a Likert-type scale and are grouped into 6 dimensions, including frequency of complaints, limitations in the activities of daily living, work-related problems, social limitations, intensity of complaints, and emotional complaints. To calculate PEmb-QoL scores, responses to individual items were transformed into a 0–100 scale, with higher scores corresponding to worse health states [16]. This required reversing the scales for questions Q1, Q4, Q5, and Q9. Scores of the constituting items for each dimension were then averaged to produce dimension scores, while an average score of all questionnaire items produced the PEmb-QoL total summary score [20]. Questions Q2 (“At what time of day are your lung symptoms most intense?”) and Q3 (“Compared to 1 year ago, how would you rate the condition of your lungs in general now?”) were considered descriptive in nature and were not scored on a Likert scale. Thus, they were interpreted as is and not incorporated into the dimension scores. Item Q4a (“Do your lung symptoms limit your daily activities at work?”) was treated as missing if a patient had chosen the “I do not work” response [16].

### 2.4 Translation

A cardiovascular disease specialist proficient in both English and Persian-translated the validated English questionnaire into Persian (forward translation). The translation was converted back into English (backward translation) by 2 different cardiovascular disease specialists proficient in both languages without reviewing the original English version. Afterward, 5 independent experts in pulmonary vascular diseases reviewed and compared the Persian and English versions to ensure clarity and to address any inconsistencies. As the final step, minor adjustments were made to the preliminary translation based on a pilot study in which 20 native Persian-speaking patients were interviewed regarding the clarity of the items after they had completed the first questionnaire draft.

### 2.5 Psychometric analysis

Psychometric analysis was conducted according to the latest standard protocols [[27], [28], [29], [30], [31], [32], [33]].

### 2.6 Acceptability and reliability

Acceptability was assessed through the completeness of data (the missing item rate). Reliability was assessed in terms of consistency between the results of repeated measurements (reproducibility) and consistency between responses within the same measurement (internal consistency). Reproducibility was reported as an intraclass correlation via the test-retest method, where the respondents were invited back to our center 3 weeks after the initial QOL assessment to retake the PEmb-QoL questionnaire. The 3-week interval was selected to prevent recall and minimize the chance of clinical changes.

Cronbach’s α is considered an adequate measure of internal consistency for individual dimensions [34]. Still, given that multidimensional constructs do not conform well with the one-dimensionality assumption of Cronbach’s α, in the current investigation, McDonald’s ω was additionally provided for the totality of the questionnaire [35,36]. The evaluation of internal consistency was completed by reporting the association between the individual items (the inter-item correlation), the association between the items and their assigned dimensions (the corrected item-total correlation), and the association between the dimensions (the domain intercorrelation). Although coefficients of internal consistency larger than 0.8 are conventionally regarded as acceptable, a more detailed interpretation should consider that these measures are also influenced by the number of items in the subscale. Supplementary Table S1, extracted from the study by Ponterotto and Ruckdeschel [37], elaborates on the acceptable thresholds for coefficients of internal consistency based on the sample size and scale length.

The score distribution, along with the floor effect and the ceiling effect, was reported for the dimensions. The quality criteria for acceptability and reliability are summarized in Supplementary Table S2. Each item was examined vis-à-vis its frequency of endorsement (respondents who selected the same item response), skewness, corrected item-total correlation with its' corresponding dimensions, and Cronbach’s α of its dimension when the item was excluded. Ideally, fewer than 25% of the items should have negative skewness, while more than 75% should have a skewness value between −1 and 1 [38]. An item is considered for removal when doing so substantially improves α or when its' item-total correlation and frequency of endorsement are outside the range of 0.2 to 0.8 [29].

### 2.7 Validity

The content validity of the PEmb-QoL has been previously investigated with respect to measurement aims, concepts, target population, and item selection [15,39].

In the present study, construct validity was investigated in terms of convergent and discriminant validity. A PRO measure is expected to have relatively high correlations with other theoretically similar PRO and non-PRO measures (ie, convergent validity) while having relatively lower correlations with theoretically dissimilar measures (ie, discriminant validity) [32,40]. Thus, we expected moderate-to-high Spearman rank correlations between the dimensions of the disease-specific PEmb-QoL and their theoretically similar counterparts from the generic SF-36 questionnaire, as well as moderate-to-high correlations between the dimension of limitations in the activities of daily living and the 6MWT results. We also expected moderate-to-low correlations between the disease-specific PEmb-QoL scores with age, sex, obesity, cancer, and other cardiovascular comorbidities.

We further evaluated internal consistency, item selection, and questionnaire structure through exploratory factor analysis (EFA). EFA investigates the coherence of item responses to suggest an underlying structure for the questionnaire. We first used the Scree test to determine the appropriate number of latent factors (ie, dimensions) and then conducted EFA via a polychoric correlation matrix with the maximum likelihood estimation method. The polychoric correlation matrix was chosen over the conventionally used Pearson correlation, given that items with fewer than 5 to 7 response options (eg, items Q4 and Q5) could violate the linearity assumption of the Pearson correlation [[41], [42], [43]]. Finally, oblique rotation (Oblimin) was applied to account for the interconnected nature of the psychological constructs and to also maximize the distinction between the extracted factors [41,44].

The R programming statistical software version 4.1.3 was used with the tidyverse, Psych, GPArotation, ggcorrplot, and Ggally packages.

## 3 Results

### 3.1 Participants

**Figure 1:**
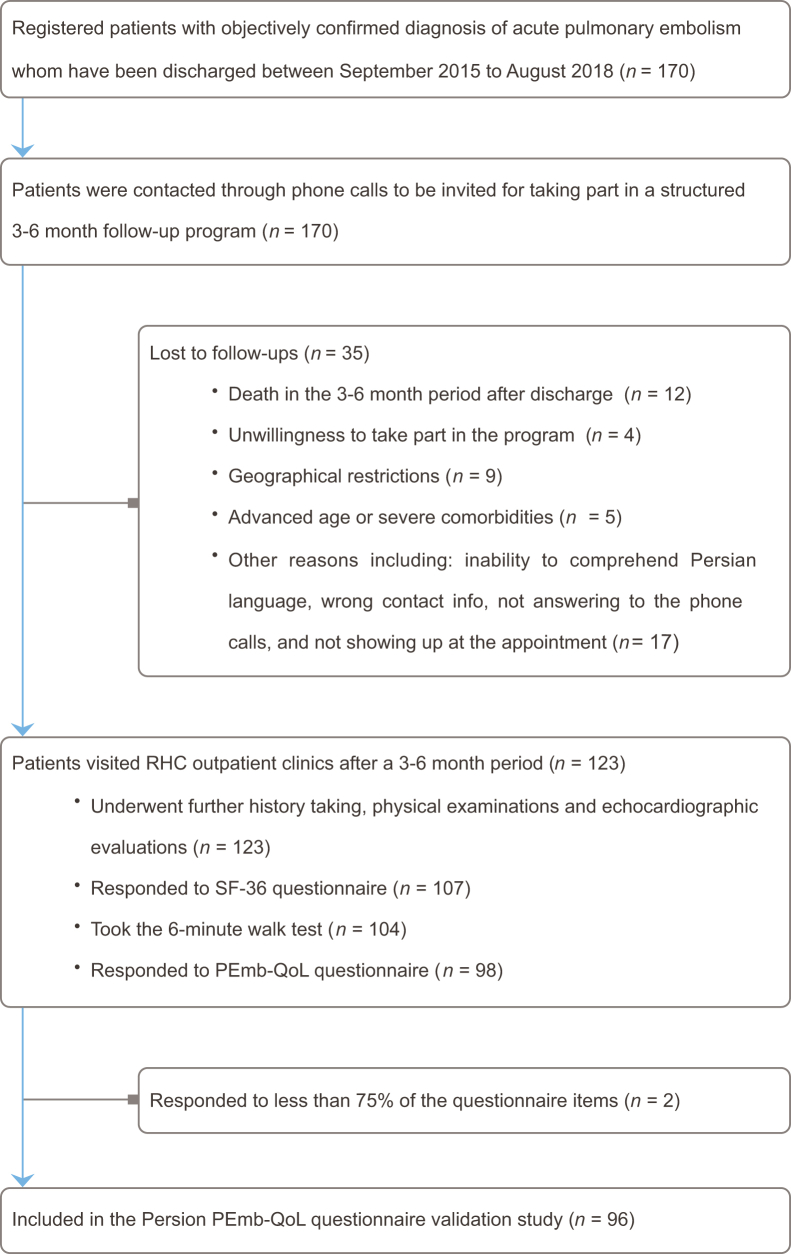
Flow diagram of exclusion criteria. PEmb-QoL, Pulmonary Embolism Quality of Life questionnaire; RHC, Rajaie Cardiovascular Medical and Research Center; SF-36, 36-item Short Form Health Survey.

Of 170 patients recruited in the RHC-PE registry, 98 agreed to complete the PEmb-QoL questionnaire. Two participants were excluded as they had responded to fewer than 75% of the items. The responses elicited from the remaining 96 participants had a minimal item missing rate (0.38%), with 6 participants having a total of 14 unresponded items. The details of the patient flow and exclusion criteria are depicted in Figure 1. Nearly half of the respondents were female. The median age of the participants was 54 years, and the obesity rate was 48%. The median (IQR) time interval between the first hospital admission due to PE and questionnaire submission was 6.1 months (IQR 5.4–7.1 months) (Table 1).

**Table 1:** Baseline characteristics of the 96 included participants.

Characteristics	N (%) or median (IQR)
Age (y)	54 (38–66)
Female	46 (48)
BMI (kg.m^-2^)	28 (25.5–31.6)
Recurrent PE	8 (8)
PE risk stratification^a^	
Low risk	25 (26)
Intermediate-low risk	21 (22)
Intermediate-high risk	31 (32)
High risk	19 (20)
Cardiopulmonary comorbidity^b^	15 (16)
Active malignancy	4 (4)
Centrally located PE^c^	42 (44)

The ethnicity of all study participants was Iranian white. BMI, body mass index; PE, pulmonary embolism.

a Risk stratification was performed based on the European Society of Cardiology guidelines for diagnosis and management of acute pulmonary embolism [22].

b Patients with a history of systolic or diastolic dysfunction and/or obstructive lung disease.

c Thrombi involving the main trunk of the pulmonary artery and/or left and right main pulmonary arteries.

### 3.2 Psychometric properties

**Figure 2:**
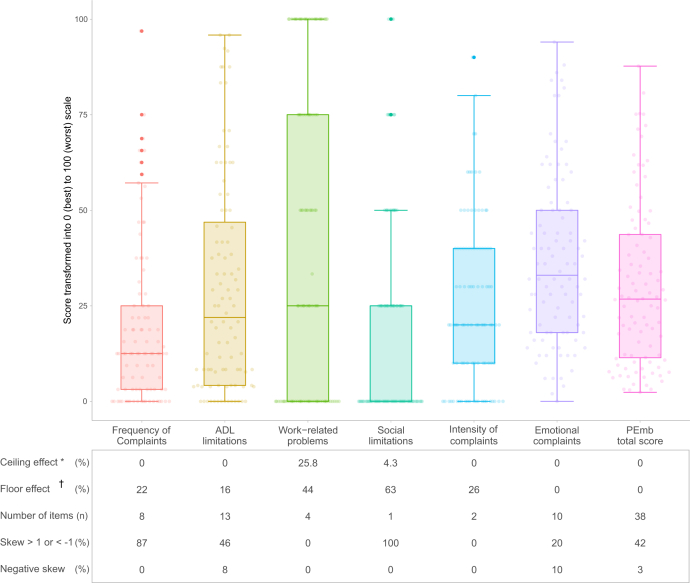
Score distribution of the PEmb-QoL total scale and subscales. Individual scores are jittered along the horizontal axis to allow visual distinction. Floor or ceiling effects <15% are desirable. Less than 25% of items should have negative skewness, and less than 25% should have a skewness outside of -1 to 1 range. ∗ Patients with the lowest possible score, ie, the best quality of life for the corresponding dimension. † Patients with the highest possible score, ie, worst quality of life for the corresponding dimension. ADL, activities of daily living; FC, frequency of complaints; PEmb-QoL, Pulmonary Embolism Quality of Life questionnaire.

Figure 2 presents the details of the score distribution for the total summary scale and dimensions of the PEmb-QoL. Although the total summary scale and the emotional complaint dimension exhibited neither a floor effect nor a ceiling effect, all the other dimensions had floor effects exceeding 15%, varying from 16.1% (for limitations in the activities of daily living) to 62.6% (for social limitations). Social limitations and work-related problems also exhibited ceiling effects of 4.3% and 25.8%, respectively.

**Figure 3:**
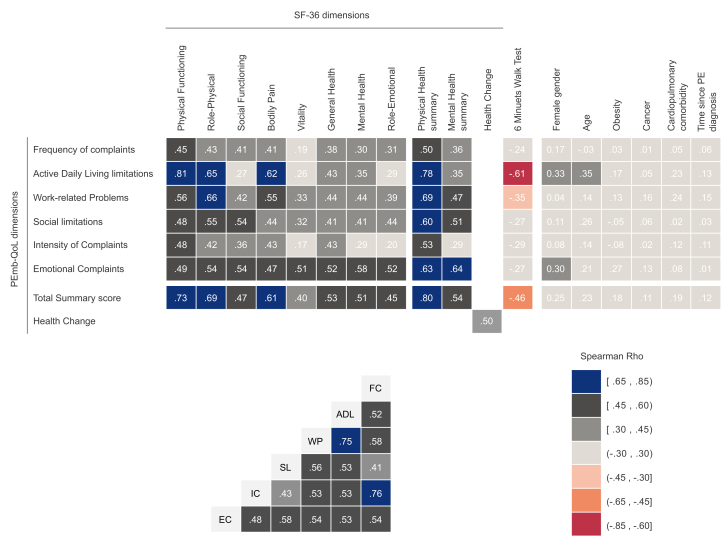
Convergent and discriminant validity (top) and domain intercorrelation (bottom) of the PEmb-QoL questionnaire. A moderate-to-high correlation between the theoretically similar dimensions is desired. PEmb-QoL, Pulmonary Embolism Quality of Life questionnaire; SF-36, 36-Item Short Form Health Survey.

Our assessment indicated excellent internal consistency reliability for the Persian version of the PEmb-QoL questionnaire (α = 0.95, ω with 3 factors = 0.96). Internal reliability was high (α ≥ 0.81) for almost all the dimensions except the intensity of complaints (α = 0.6). All dimensions had appropriate mean-corrected item-total correlations, ranging between 0.43 and 0.71 (Table 2). Moreover, all PEmb-QoL dimensions showed moderate-to-strong domain intercorrelations. The weakest correlation was between the intensity of complaints and social limitations (*r* = 0.43) and, expectedly, the strongest between limitations in the activities of daily living limitations and work-related problems (*r* = 0.75) as well as between the frequency of complaints and the intensity of complaints (*r* = 0.76) (Figure 3).

**Table 2:** Assessment of Internal reliability, discriminative ability, redundancy, and homogeneity.

PEmb-QoL dimension	PEmb-QoL questions	Number of items	Cronbach’s α	Mean inter-item correlation	Mean-corrected item-total correlation
Frequency of complaints	Q1	8	0.83	0.38	0.56
ADL limitations	Q4	13^a^	0.94	0.53	0.70
Work-related problems	Q5	4	0.86	0.62	0.71
Social limitations	Q6	1	—	—	—
Intensity of complaints	Q7,8	2	0.6	0.4	0.38
Emotional complaints	Q9	10	0.81	0.3	0.48
Pemb-QoL questionnaire	Q1,4,5,6,7,8,9	37^a^	0.95	0.33	0.54

ADL, activities of daily living; PEmb-QoL, pulmonary embolism quality of life.

a The PEmb-QoL questionnaire contained 38 Likert-type items; however, item Q4a was omitted from the reliability assessment due to the high number of participants with an “I do not work” response which was treated as a missing value. Q2 and Q3 are not Likert-type scales and were thus not included in the reliability analysis.

Supplementary Figure S2 depicts the response distribution for individual items. Negative skewness was observed only with items 4d (skewness −0.41) and 9d (skewness −0.08). However, only 22 scored items (58%) had skewness between −1 and 1. Item Q1a (“Pain behind or between the shoulder blades?”) had the lowest item-total correlation (*r* = 0.34). This item was also the only item that, upon removal, would improve the internal consistency of its designated dimension (α = 0.83 to α = 0.85).

Of the 96 included participants, 25 (26%) returned to complete the questionnaire for the second time. Compared to the primary respondent group, participants of the retest were, on average younger (55 years old vs 46.5 years old, *P* value = .02) and had more odds of being male (odds ratio = 2.85, *P* value = .05). The test-retest analysis indicated moderate-to-high intraclass correlations for all dimensions, ranging from 0.70 for work-related problems to 0.97 for the intensity of complaints (Table 3). The results of the acceptability and reliability analysis are summarized in Supplementary Table S2.

**Table 3:** Test-retest reliability analysis with 25 participants.

PEmb-QoL dimension	Intraclass correlation coefficient (95% confidence interval)
Frequency of complaints	0.97 (0.92–0.99)
Activities of daily living limitations	0.98 (0.95–0.99)
Work-related problems	0.98 (0.96–0.99)
Social limitations	0.96 (0.91–0.99)
Intensity of complaints	0.92 (0.83–0.97)
Emotional complaints	0.97 (0.93–0.99)

**Figure 4:**
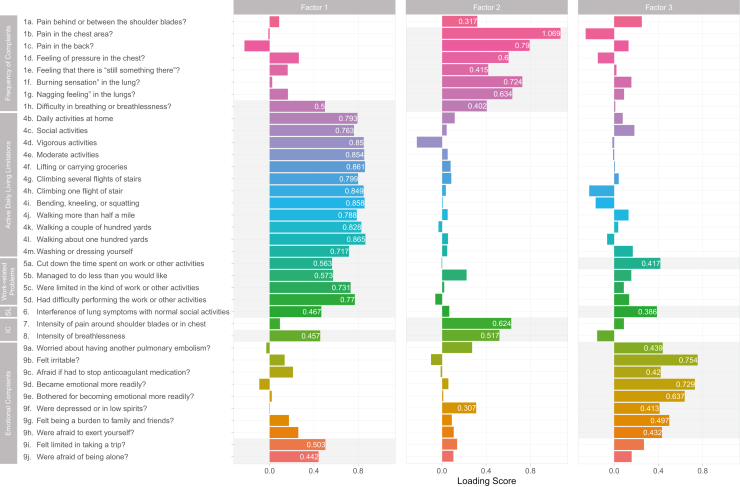
Exploratory factor analysis (EFA) of the Persian version of the PEmb-QoL questionnaire with Oblimin rotation method. EFA uses a covariance matrix to extract a set of latent common variables that best explain the observed variance in the responses to questionnaire items. The 3 extracted factors accounted for 34%, 15%, and 11% of the total variance in patient responses. Factor loadings represent the regression coefficient of each item. Coefficients <0.35 were not mentioned. IC, intensity of complaint; PEmb-QoL, Pulmonary Embolism Quality of Life questionnaire; SL, social limitations.

Item 4a (“Daily activities at work”) was excluded from the EFA as the majority of the participants (65%) had chosen the “I do not work” response. The EFA yielded 3 latent factors, cumulatively accounting for 60% of the total observed variances (Figure 4). Factor 1 contained all items of activities of daily living limitations and work-related problems (4b-m and 5a-d). Factor 1 also incorporated item 1h, item 8 (“concerning the frequency and intensity of breathlessness”), item 6 (“Interference of lung symptoms with normal social activities?”), item 9i (“Felt limited in taking a trip?”), and item 9j (“Afraid of being alone?”). Factor 2 comprised items from the frequency of complaints (1b-g) and the intensity of complaints (item 7 and 8). Factor 3 contained item 5a, 6, and 9b-h. Item 1a (“Pain behind or between the shoulder blades?”) had poor loading values across the board, whereas items 1h, 6, and 8 had high loading values in more than a single factor. These cross-loadings persisted despite the application of different rotation methods.

The details of convergent and discriminant validity are presented in Figure 3. The PEmb-QoL dimensions of frequency of complaints, the intensity of complaints, activities of daily living limitations, and work-related problems had stronger associations with the physical health component of the SF-36 questionnaire. Nonetheless, emotional complaints had moderate correlations with all SF-36 dimensions, varying from bodily pain (*r* = 0.47) to mental health (*r* = 0.58), which resulted in a roughly similar association with mental health (*r* = 0.64) and physical health summary scores (*r* = 0.63) of the SF-36. The median (IQR) distance walked during the 6MWT was 390 meters (320–450 m). Limited exercise capacity, defined as distances less than 350 meters, was observed in 39% of the participants. Expectedly, the 6MWT distance converged best with the scores from limitations in the activities of daily living (*r* = −0.61) and work-related problems (*r* = −0.35).

## 4 Discussion

To our knowledge, The present study is the first, to translate and validate a PE-specific PRO instrument in a Middle Eastern country. Cross-cultural validation of the PEmb-QoL questionnaires in Persian-speaking populations would allow researchers to study the impact of PE and its treatments on the quality of life of a large population of PE survivors, given that over 120 million people worldwide speak the Persian language. Based on our results, almost all the dimensions proposed by the original validation study [15] yielded appropriate inter-item correlations, corrected item-total correlations, domain intercorrelations, and high-reliability coefficients. Our analysis also supported the convergent and discriminant validity of the PEmb-QoL dimensions in the Persian population.

Overall, the PEmb-QoL total summary scale and emotional complaints conformed to almost all gold reliability standards, and their appropriate inter-item correlations and absence of ceiling or floor effects indicated their adequate discriminative power. Still, 5 out of the 6 dimensions had floor effects, with work-related problems also exhibiting a ceiling effect. Similar findings have been observed in all other validation studies of the PEmb-QoL questionnaire [[16], [17], [18], [19], [20]]. These floor and ceiling effects can have several nonmutually exclusive explanations. For one, the substantial floor effects could mean that for many patients, these 5 dimensions of quality of life took no toll due to PE. Two other frequently cited explanations for the undesirable floor effects are gradual health improvements prior to QOL assessments and social desirability bias toward better health status [16,17,20]. Although these 2 explanations are well-substantiated, they were proposed by studies involving European cohorts with relatively long intervals (median 15–43 months) between the acute PE event and questionnaire completion, which could have allowed for gradual health improvements. Nevertheless, the fact that similar results were observed in cross-cultural validation studies in non-European populations with much shorter PE-to-questionnaire completion intervals—a median of 6.1 months in the current study and a median of 4.5 months in the Chinese validation [19]—indicates that other possibilities should also be considered. If resulted from measurement biases, floor and ceiling effects could potentially imply that the instrument struggles to distinguish respondents at the extreme ends of the scale (ie, the best/worst score), which could, in turn, limit the ability of the instrument to detect small changes in the health of these patients in response to effective interventions. Scales with few items, especially those containing few response options or unbalanced response sets, are prone to the undesirable floor and ceiling effects [45]. As a case in point, the marked floor effect of social limitations could be explained by the fact that it is created by a single item (Q6). On the other hand, work-related problems (Q5a-d) constitute the only dimension exhibiting both floor and ceiling effects, possibly because it exclusively comprised items with dichotomous response categories (Supplementary Figure S2).

After clarifying content validity in terms of measured concepts, Klok et al. [15] structured dimensions through clinical concepts and patient interviews for the current version of the questionnaire. However, the suggested structure should be bolstered through exploratory factor analysis before the confirmatory process [8,12]. Our EFA suggested a 3-factor structure closely resembling the study of the French population [17]. These factors were, by and large, combinations of highly associated dimensions. Factor 1 (Q1h, Q4b-m, Q5a-d, Q9i, Q9j) was predominantly formed by items of dimension activities of daily living limitations and work-related problems, factor 2 (Q1b-g, Q7, Q8) was formed by items of the frequency of complaints and intensity of complaints, whereas factor 3 was largely formed by items from emotional complaints. Henceforth, we regard these 3 factors as functional, symptom, and emotional components, respectively.

As suggested by the FDA guidelines [12], we complemented the Klok et al. [15] theoretical framework for item selection through factor, reliability, and item response analyses. Item 1a (“Pain behind or between the shoulder blades?”) was removed because of its poor loading values across all 3 extracted factors. This item also had the lowest coherence with its originally designated dimension (corrected item-total *r* = 0.38). The removal of item 1a is supported by the fact that the presented description of “pain behind or between the shoulder blades” is not only an uncommon, chronic complication of PE, but it could also overlap with musculoskeletal complaints [46,47]. Four items showed cross-loading. Items 1h (“Difficulty in breathing or breathlessness?”) and 8 (“Intensity of breathlessness?”) had significant loadings in both functional and symptom components, while item 5a (“Cut down the time spent on work or other activities?”) and 6 (“Interference of lung symptoms with normal social activities?”) had significant loadings in both functional and emotional components. Furthermore, although items 9i (“Felt limited in taking a trip?”) and 9j (“Afraid of being alone?”) were originally assigned to emotional complaints, they clustered better with the items of the functional component rather than with the emotional component. A logical explanation could be that individuals tend to judge their independence, at least in part, by their self-perception of physical performance.

Mazdak Tavoly et al. [20] was the first to report and evaluate the total score for the PEmb-QoL questionnaire. Since then, validation studies, including the present one, have found this scale to enjoy adequate reliability [[15], [16], [17], [18], [19], [20],^48^]. Nonetheless, we believe that the validity of a total summary scale should be revisited. It is generally discouraged to calculate a single total summary score for multidimensional constructs such as QOL, as this process intrinsically involves assigning unjustified weights to each dimension [32,49]. In this case, the Norwegian study calculated a single total summary score by averaging dimensional scores, whereas the French, German, and Chinese studies calculated it by averaging individual item scores. While the former approach assumes equilibrium between the contributions of all dimensions toward QOL, the latter implicitly assumes more weight for dimensions with more items. The rationale behind these assumptions, however, is unclear. Even though the dimensions of QOL are highly influenced by one another, the preference weight for each dimension (eg, physical well-being and social well-being) may vary between individuals. Conflating the scores of these dimensions without incorporating their preference weight for each respondent could, therefore, be unwarranted [49]. We also cannot confirm the reliability or validity of measuring social limitations solely based on item Q6, primarily because single-item dimensions generally fail not only to sufficiently capture all aspects of a general concept, but also to qualify for the conventional analysis of reliability [12]. The cross-loading values of items Q6 (“Interference of lung symptoms with normal social activities”) and Q5a (“Cut down the time spent on work or other activities?”) also demonstrate that they should be interpreted as part of the shared attributes between broader concepts rather than being assigned to, or comprehensively describe, a single subscale.

Our study is subject to several limitations. First, of the 170 registered patients in the RHC-PE registry, only 98 (58%) participated in the PEmb-QoL evaluation (Figure 1). Of these primary respondents, only 25 (26%) returned in the following 2–4 weeks acceptable window of retest analysis. The low turnout for the follow-up sessions precluded an evaluation of the questionnaire’s responsiveness. Second, a higher median age in the nonparticipant group (60 vs 54 years old, *P* value = 0.03) and the fact that 12 patients died prior to study enrollment indicate that older and probably sicker patients may have been underrepresented in our study population. We believe this low turnout to be the result of our preferred mode of questionnaire administration; during their 6 months follow-up session, patients were asked to complete the questionnaires in the presence of an experienced nurse to minimize missing item responses, and accommodate low literacy, visually impaired, and elderly patients [14]. An in-person mode of administration also allowed us to incorporate the results of 6MWT into our study as a non-PRO measure of functional performance. However, given that our high-volume tertiary cardiovascular center receives patients nationwide, geographical restrictions made patient recruitment, in-person follow-ups and retest sessions challenging.

In summary, the Persian version of the PEmb-QoL questionnaire is a psychometrically valid and reliable PRO instrument to assess health-related QOL in patients with a history of acute PE. We extracted a 3-component structure comprising functional (items 1h, 4b-5d, 6, 8, 9i, and 9j), symptom (1b-h, 7, and 8), and emotional (5a, 6, and 9a-h) components. Further longitudinal studies are warranted to evaluate responsiveness and establish the minimum important difference.
