# Supplementary figures and images for: Cross-cultural validity of the Pulmonary Embolism Quality of Life questionnaire in the quality of life survey after pulmonary embolism: A Persian-speaking cohort
Source: Res Pract Thromb Haemost. 2023 Apr 1;7(3):100145. doi: 10.1016/j.rpth.2023.100145 (PMC10163673; doi:10.1016/j.rpth.2023.100145)

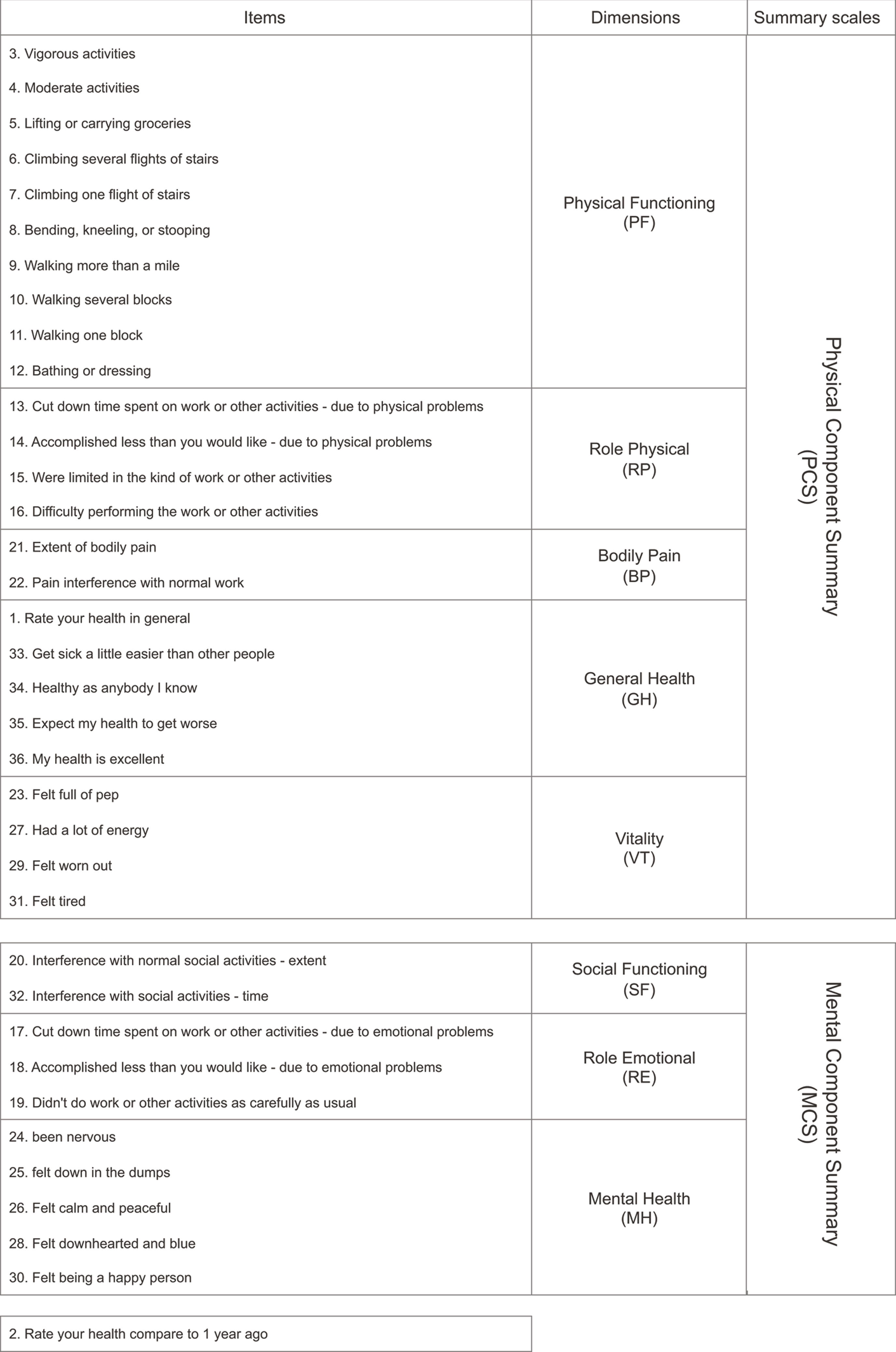

Supplement: Supplementary Figure S1 [file figs1.jpg]

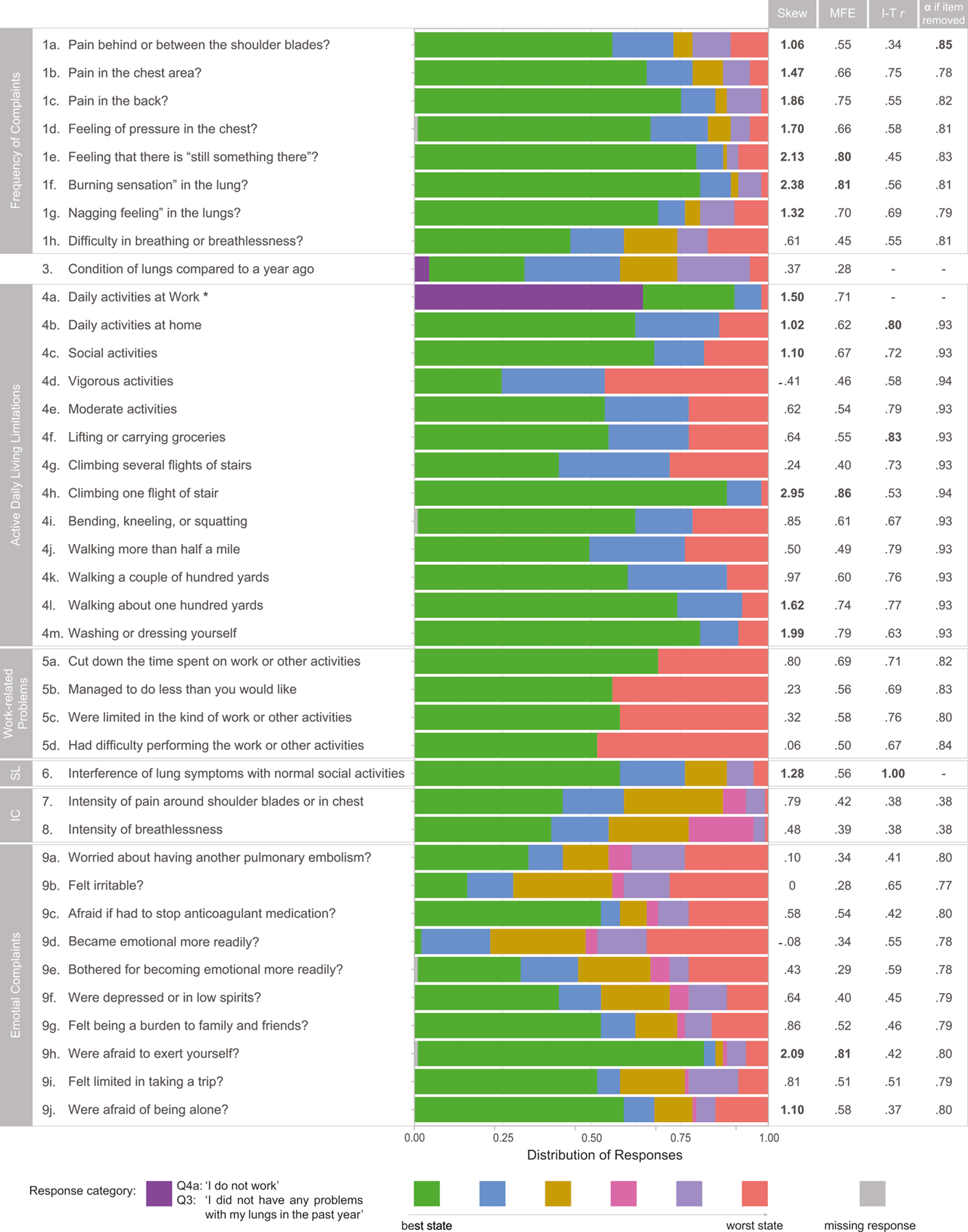

Supplement: Supplementary Figure S2 [file figs2.jpg]
