# Supplementary material for: Cross-cultural validity of the Pulmonary Embolism Quality of Life questionnaire in the quality of life survey after pulmonary embolism: A Persian-speaking cohort
Source: Res Pract Thromb Haemost. 2023 Apr 1;7(3):100145. doi: 10.1016/j.rpth.2023.100145 (PMC10163673; doi:10.1016/j.rpth.2023.100145)
Supplement: Supplementary Material [file mmc1.docx]

# Supplemental materials

**Table S1.** Matrix for estimating adequacy of internal consistency coefficients with research measure

|  |  | **Sample size** | | |
| --- | --- | --- | --- | --- |
| **Item per subscale** | **Rating** | **N < 100** | **N = 100-300** | **N > 300** |
| ≤ 6 | Excellent | .75 | .80 | .85 |
|  | Good | .70 | .75 | .80 |
|  | Moderate | .65 | .70 | .75 |
|  | Fair | .60 | .65 | .70 |
|  |  |  |  |  |
| 7-11 | Excellent | .80 | .85 | .90 |
|  | Good | .75 | .80 | .85 |
|  | Moderate | .70 | .75 | .80 |
|  | Fair | .65 | .70 | .75 |
|  |  |  |  |  |
| ≥ 12 | Excellent | .85 | .90 | .95 |
|  | Good | .80 | .85 | .90 |
|  | Moderate | .85 | .90 | .95 |
|  | Fair | .70 | .75 | .80 |
| Note. ‒ internal consistency coefficient falling below the "Fair" rating for its particular cell would be deemed "Unsatisfactory.” | | | | |
| This table was excerpted from the study by Pontekotto & Ruckdeschel [37]. | | | | |

| **Table S2**.The quality criteria and summary results of acceptability and reliability | | | | | | | | |
| --- | --- | --- | --- | --- | --- | --- | --- | --- |
| **Term** | Quality criteria | PEmb | FC | ADL | WP | SL | IC | EC |
| Completeness of data | Item missing rate < 10% | ✅ | ✅ | ✅ | ✅ | ✅ | ✅ | ✅ |
| Internal consistency | Refer to Table S1 | ⬆️ | ✅ | ✅ | ✅ | - | 🟥 | ✅ |
|  | 0.2 < Average inter-item *r* < 0.5 | ✅ | ✅ | ⬆️ | ⬆️ | - | ✅ | ✅ |
|  | Average item-total *r >* 0.4 | ✅ | ✅ | ✅ | ✅ | - | ✅ | ✅ |
| Reproducibility | Test-retest ICC _consistency_ > 0.9 | ✅ | ✅ | ✅ | ✅ | - | ✅ | ✅ |
| Ceiling effect | Cases with highest possible score < 15% | ✅ | ✅ | ✅ | 🟥 | ✅ | ✅ | ✅ |
| Floor effect | Cases with lowest possible score < 15% | ✅ | 🟥 | 🟥 | 🟥 | 🟥 | 🟥 | ✅ |
| Acceptable item total correlation for individual items is in the range of 0.2-0.8  ✅ indicates that measure conforms to the criterion, ⬆️ indicates that the measure exceeds the upper bound of the criterion range. Excessively high values for Cronbach’s α, inter-item correlation and item-total correlation are not signs of diminished reliability but may indicate redundancy, 🟥 indicates that the measure does not conform to the quality criterion. | | | | | | | | |
